# *Staphylococcus aureus* susceptibility to complestatin and corbomycin depends on the VraSR two-component system

**DOI:** 10.1128/spectrum.00370-23

**Published:** 2023-08-30

**Authors:** Carmen Gómez-Arrebola, Sara B. Hernandez, Elizabeth J. Culp, Gerard D. Wright, Cristina Solano, Felipe Cava, Iñigo Lasa

**Affiliations:** 1 Laboratory of Microbial Pathogenesis, Navarrabiomed, Hospital Universitario de Navarra (HUN), Universidad Pública de Navarra (UPNA), IdiSNA, Pamplona, Spain; 2 Department of Molecular Biology, Laboratory for Molecular Infection Medicine Sweden, Umeå Centre for Microbial Research, Umeå University, Umeå, Sweden; 3 Department of Biochemistry and Biomedical Sciences, M. G. DeGroote Institute for Infectious Disease Research, David Braley Centre for Antibiotic Discovery, McMaster University, Hamilton, Ontario, Canada; Riverside University Health System, Medical Center -University of California, Riverside, California, USA

**Keywords:** *Staphylococcus aureus*, two-component system, VraSR, complestatin, corbomycin, autolysins

## Abstract

The overuse of antibiotics in humans and livestock has driven the emergence and spread of antimicrobial resistance and has therefore prompted research on the discovery of novel antibiotics. Complestatin (Cm) and corbomycin (Cb) are glycopeptide antibiotics with an unprecedented mechanism of action that is active even against methicillin-resistant and daptomycin-resistant *Staphylococcus aureus*. They bind to peptidoglycan and block the activity of peptidoglycan hydrolases required for remodeling the cell wall during growth. Bacterial signaling through two-component transduction systems (TCSs) has been associated with the development of *S. aureus* antimicrobial resistance. However, the role of TCSs in *S. aureus* susceptibility to Cm and Cb has not been previously addressed. In this study, we determined that, among all 16 *S. aureus* TCSs, VraSR is the only one controlling the susceptibility to Cm and Cb. Deletion of *vraSR* increased bacterial susceptibility to both antibiotics. Epistasis analysis with members of the vraSR regulon revealed that deletion of spdC, which encodes a membrane protein that scaffolds SagB for cleavage of peptidoglycan strands to achieve physiological length, in the vraSR mutant restored Cm and Cb susceptibility to wild-type levels. Moreover, deletion of either *spdC* or *sagB* in the wild-type strain increased resistance to both antibiotics. Further analyses revealed a significant rise in the relative amount of peptidoglycan and its total degree of cross-linkage in *ΔspdC* and *ΔsagB* mutants compared to the wild-type strain, suggesting that these changes in the cell wall provide resistance to the damaging effect of Cm and Cb.

**IMPORTANCE:**

Although *Staphylococcus aureus* is a common colonizer of the skin and digestive tract of humans and many animals, it is also a versatile pathogen responsible for causing a wide variety and number of infections. Treatment of these infections requires the bacteria to be constantly exposed to antibiotic treatment, which facilitates the selection of antibiotic-resistant strains. The development of new antibiotics is, therefore, urgently needed. In this paper, we investigated the role of the sensory system of *S. aureus* in susceptibility to two new antibiotics: corbomycin and complestatin. The results shed light on the cell-wall synthesis processes that are affected by the presence of the antibiotic and the sensory system responsible for coordinating their activity.

## INTRODUCTION

The indiscriminate use of antibiotics has led to the emergence of numerous multidrug-resistant (MDR) bacteria (1 – 5). Two main actions to combat MDR are rigorous policy interventions ensuring the judicious use of antibiotics and a renewal effort for discovering new antimicrobial substances. Recently, two members of a new functional class of natural glycopeptide antibiotics produced by *Streptomyces* have been described (6). Both antibiotics, named complestatin (Cm) and corbomycin (Cb), are low molecular weight linear peptides biosynthesized by nonribosomal peptide synthetases. The mechanism of action of both Cm and Cb is based on their ability to bind the peptidoglycan (PG), the structural unit of the bacterial cell wall. Such binding blocks the access of autolysins, enzymes required to break down the peptidoglycan for the insertion of new precursors during peptidoglycan growth and for cell separation after division (7). Autolysins are a highly redundant family of enzymes with hydrolytic functions that include: (i) amidases that cleave the amide bond between *N*-acetylmuramic (NAM) acid and the L-alanine residue at the N-terminal of the stem peptide, (ii) glycosidases that cleave the glycosidic linkages between *N*-acetylmuramic acid and *N*-acetylglucosamine (NAG), and (iii) peptidases that cleave amide bonds between amino acids within the peptidoglycan chain (8, 9). The finding that Cm and Cb impact the activity of a broad range of autolysins instead of selectively targeting a specific type is relevant because inhibition of a single enzyme is usually compensated by other members of the family (10, 11). Peptidoglycan degradation must be tightly regulated and constantly balanced with peptidoglycan synthesis to maintain cell-wall homeostasis (12, 13). Many bacteria use two-component systems (TCSs) that allow bacteria to sense and respond to changes in the environment (14, 15) to modulate peptidoglycan biosynthesis and remodeling in response to a variety of signals, including the presence of cell-wall-acting antibiotics (16 – 18). Presently, there is no comprehensive knowledge of whether and how signal transduction pathways are involved in Cm and Cb sensitivity. However, such knowledge will be valuable to uncover differences in peptidoglycan remodeling under different environmental conditions and anticipate how antibiotic resistance might emerge (6).

One pathogen for which it is necessary to develop new antibiotics because of its alarmingly high resistance levels to current antimicrobials is *Staphylococcus aureus* (19, 20), a Gram-positive, coagulase-positive, and non-motile bacterium responsible for causing life-threatening infections including endocarditis, sepsis, osteomyelitis, and pneumonia (21). One-third of the general population is colonized by *S. aureus*, constituting a risk factor for infection by this pathogen (22). Importantly, both Cm and Cb are active against methicillin-resistant (MRSA), vancomycin-intermediate, and daptomycin-resistant *S. aureus* strains (6). In *S. aureus*, the activity and/or expression of several autolysins is controlled by various TCSs (23 – 25). Furthermore, different TCSs of *S. aureus* have already been implicated in MDR through modification of the cell wall, efflux mechanisms, and inhibition of drug uptake (26, 27): WalKR and VraSR play a role in the resistance to vancomycin (28 – 31), BraRS affects the susceptibility to bacitracin and nisin (32), GraRS affects the susceptibility to cationic antimicrobial peptides (33), ArlRS regulates resistance to ceftaroline (34), and HptSR is related with resistance to fosfomycin (35). The absence of any of these TCSs increases susceptibility to the corresponding antibiotic. Thus, they represent targets whose inhibition might have antimicrobial activity or may resensitize *S. aureus* to currently ineffective antibiotics.

The present work addresses whether any TCS network of *S. aureus* is associated with the susceptibility to Cm and Cb. To this end, we have interrogated a collection of 15 *S. aureus* mutants in each TCS as well as a mutant lacking all 15 non-essential TCSs and its derivatives containing a single TCS for their susceptibility to Cm and Cb (24, 36). Our analyses identified VraSR as the only TCS involved in *S. aureus* susceptibility to both antibiotics. Exploration of specific VraSR-regulated proteins involved in cell-wall remodeling determined that the absence of the autolysin SagB or its interacting partner SpdC increases resistance to Cm and Cb. Subsequent characterization of the peptidoglycan composition suggested that such a rise in resistance might be due to a significant increase in the relative amount of peptidoglycan per optical density (OD) of culture and its degree of cross-linkage identified in Δ*sagB* and Δ*spdC* mutant cells.

## MATERIALS AND METHODS

### Bacterial strains, plasmids, oligonucleotides, and culture conditions

Bacterial strains, plasmids, and oligonucleotides used in this work are listed in Tables S1, S2 and S3, respectively. *Escherichia coli* and *S. aureus* strains were routinely grown in Luria-Bertani medium (Conda-Pronadisa), Trypticase soy broth (TSB; Conda-Pronadisa), or Mueller Hinton broth (MHB; Conda-Pronadisa) at 37°C. When required, media were supplemented with appropriate antibiotics at the following concentrations: erythromycin, 10 µg mL^−1^; ampicillin, 100 µg mL^−1^; and chloramphenicol, 20 µg mL^−1^. Bacteriological agar was used as gelling agent (VWR International).

### DNA manipulations

Routine DNA manipulations were performed using standard procedures unless otherwise indicated. Oligonucleotides were synthesized by StabVida (Caparica, Portugal). FastDigest restriction enzymes, Phusion DNA polymerase, and the Rapid DNA Ligation Kit (Thermo Scientific) were used according to the manufacturer’s instructions. Plasmids were purified using a Macherey-Nagel Plasmid Purification Kit according to the manufacturer’s protocol. Plasmids were transformed in *E. coli* by electroporation (1 mm cuvette; 200 Ω, 25 µF, 1,250 V; Gene Pulser X-Cell electroporator, BioRad). *S. aureus* competent cells were generated as previously described (37). Plasmids were transformed in *S. aureus* by electroporation (1 mm cuvette; 100 Ω, 25 µF, 1,250 V; Gene Pulser X-Cell electroporator, BioRad). All constructed plasmids were confirmed by Sanger sequencing at StabVida (Caparica, Portugal).

### Gene deletion and *mgt* overexpression

Generation of deletion mutants was performed as described in reference (38) with some modifications. Briefly, two fragments of at least 500 bp that flanked the left and right of the gene targeted for deletion (*ssaA*, *isaA*, *spdC*, and *sagB*) were amplified by PCR using primers AB and CD (Table S3) and chromosomal DNA from *S. aureus* MW2 as a template. PCR products (AB and CD fragments) were purified and used as templates in an overlapping PCR carried out with primers AD (Table S3). The corresponding PCR products were cloned into pJET 1.2 Blunt vector and then subcloned into the shuttle vector pMAD (38), generating plasmids pMAD::*ssaA*, pMAD::*isaA*, pMAD::*spdC*, and pMAD::*sagB*. pMAD plasmids were purified from *E. coli* IM01B and transformed into the MW2 wild type or Δ*vraSR* mutant strain by electroporation. Homologous recombination experiments were performed as described (38). Erythromycin-sensitive white colonies, which did not further contain the pMAD plasmid, were tested by PCR using primers D and E (Table S3).

To construct the pCN51::*mgt* plasmid, the *mgt* gene was amplified by PCR with primers *mgt*_Fw and *mgt*_Rv (Table S3) and chromosomal DNA from *S. aureus* MW2 as a template. The PCR product was purified and cloned into pJET 1.2 Blunt vector and then subcloned into plasmid pCN51 (39) digested with *BamH*I and *EcoR*I. The resulting plasmid, pCN51::*mgt*, was purified from *E. coli* IM01B and transformed into the Δ*vraSR* mutant strain by electroporation.

### Construction of ΔXV derivatives expressing a single TCS

Construction of pCN51 plasmids expressing a complete *hptRS*, *lytRS*, *saeRS*, *tcs7RS*, *phoRP*, *airRS*, *agrRS*, *kdpDE*, *hssRS*, or *braRS* TCS (histidine kinase and response regulator encoding genes) was performed as described in reference (36) with primers shown in Table S3.

### Antibiotic susceptibility testing

The minimum inhibitory concentration (MIC) was determined following the EUCAST (European Commitee on Antimicrobial Susceptibility Testing) reading guide for broth microdilution. Briefly, twofold dilutions of antibiotics in U-shaped bottom 96-well microplates (TC Microwell 96U Nunclon, Thermo Fisher) containing MHB were prepared. Overnight cultures of the bacteria were adjusted to 5 × 10^5^ CFU mL^−1^, and 100 µL was used to inoculate the same volume of MHB containing the antibiotics. Plates were incubated at 37°C for 24 h, and the lowest concentration inhibiting visible bacterial growth was recorded as MIC. To assess antibiotic susceptibility over time at a specific antibiotic concentration, overnight cultures of bacterial strains were diluted to an optical density at 595 nm (OD_595_) of 0.1 in fresh MHB medium. A volume of 5 µL of the adjusted cultures was used to inoculate 195 µL of MHB (three replicates of each) containing complestatin, corbomycin, or bacitracin at the desired concentration, using 96-well plates (Thermo Scientific). Growth kinetics were assayed using a Synergy H1 hybrid multimode microplate reader (Biotek). Growth data (OD_595_) were collected every 30 min for 20 h at 37°C with shaking (fast orbital shaking; 425 rpm). MHB was supplemented with 0.1 µg mL^−1^ of anhydrotetracycline to induce gene expression from the pRMC2 plasmid. In the case of strains carrying the pCN51 inducible plasmid, experiments were carried out without cadmium supplementation since pCN51 shows a basal expression in the absence of cadmium. *In vitro* evolutionary studies to select mutants resistant to the antibiotics were performed as previously described (6).

### Peptidoglycan isolation and analysis

To purify the PG of the strains under study, an established protocol was followed with some modifications (40). Briefly, 200 mL cultures were grown to an OD_595_ of 0.5, and cell pellets were mechanically lysed using glass beads (0.1 mm diameter) and a Mini-Beadbeater. The supernatants were ultracentrifuged, and resulting pellets were resuspended in 1 mL Tris-HCl 100 mM pH 7.5 and treated with 40 µL MgSO_4_ 1 M, 2 µL RNase A (500 µg mL^−1^), and 1 µL DNase I (100 µg mL^−1^) for 2 h and then with 50 µL CaCl_2_ and 100 µL trypsin (2 mg mL^−1^) overnight. Samples were centrifuged at 14,000 rpm, and the pellet was treated with 1 mL LiCl 8 M, 1 mL EDTA (ethylene diamine tetra-acetic acid) 100 mM, and 1 mL of acetone. After three washes with cold MilliQ water, pellets were resuspended in 1 mL of hydrofluoric acid 48% and incubated with shaking during 48 h at 4°C to remove teichoic acids. Finally, pellets were washed four times with cold MilliQ water and resuspended in phosphate buffer 50 mM pH 4.9. PGs were digested with 4 µL muramidase (mutanolysin from *Streptomyces globisporus* ATCC 21553 at 1 mg mL^−1^) for 24 h at 37°C. Before the analysis by liquid chromatography, digested PGs were reduced as previously described using borate buffer 0.5 M pH 9 and NaBH_4_ during 30 min. Finally, the pH of the samples was adjusted to 3 by addition of 25% orthophosphoric acid. Peptidoglycan profiles were obtained by separating the different muropeptides using a UPLC (Ultra Performance Liquid Chromatography) system (Waters Corp.). The previously described organic method (41) was modified to achieve a better separation of the peaks changing the percentage of buffer A for the gradient as follows: 98% at minutes 0 to 1; 90% at minutes 15 to 15.10; 85% at minutes 18 to 18.10; 0% at 37 min; and 98% at min 20.10 to 25. Muropeptides were detected by measuring the absorbance at 204 nm.

Peak identities were assigned by mass spectrometry using an UPLC system coupled to a Xevo G2/XS Q-TOF mass spectrometer (Waters Corp.). Chromatographic separation and mass analysis were performed as previously described (42) using an ACQUITY UPLC BEH C18 Column (Waters Corp.) heated at 45°C. An amount of 0.1% formic acid in Milli-Q water and 0.1% formic acid in acetonitrile were used as eluents. The QTOF-MS (quadrupole time-of-flight mass spectrometry) instrument was operated in positive ionization mode, and MS^e^ was performed for acquisition of the data using the following parameters: capillary voltage at 3.0 kV, source temperature to 120°C, desolvation temperature to 350°C, sample cone voltage at 40 V, cone gas flow 100 L/h, desolvation gas flow 500 L/h, and collision energy (CE); low CE: 6 eV and high CE ramp: 15–40 eV. Mass spectra were acquired at a speed of 0.25 s/scan. The scan was in a range of 100–2,000 m/z. Data acquisition and processing were performed using UNIFI software package (Waters Corp.).

Structural characterization of muropeptides was determined based on their mass spectrometric data and tandem mass spectrometric fragmentation pattern, matched with peptidoglycan composition and structure reported previously (43, 44). Relative amount of each muropeptide was calculated by dividing the peak area of a muropeptide by the total area of the chromatogram. The relative amount of peptidoglycan per OD of culture was calculated by integrating the total area of the chromatogram obtained from cell cultures normalized to the OD_595_, and the degree of cross-linkage comparing the area of all cross-linked muropeptides (dimers, trimers, tetramers, pentamers, and hexamers) with the total area of the chromatogram. All peptidoglycan analyses were performed using biological triplicates, and a representative chromatogram is shown for each strain.

## RESULTS

### The VraSR TCS controls the susceptibility of *S. aureus* to the antibiotics Cm and Cb

**Fig 1:**
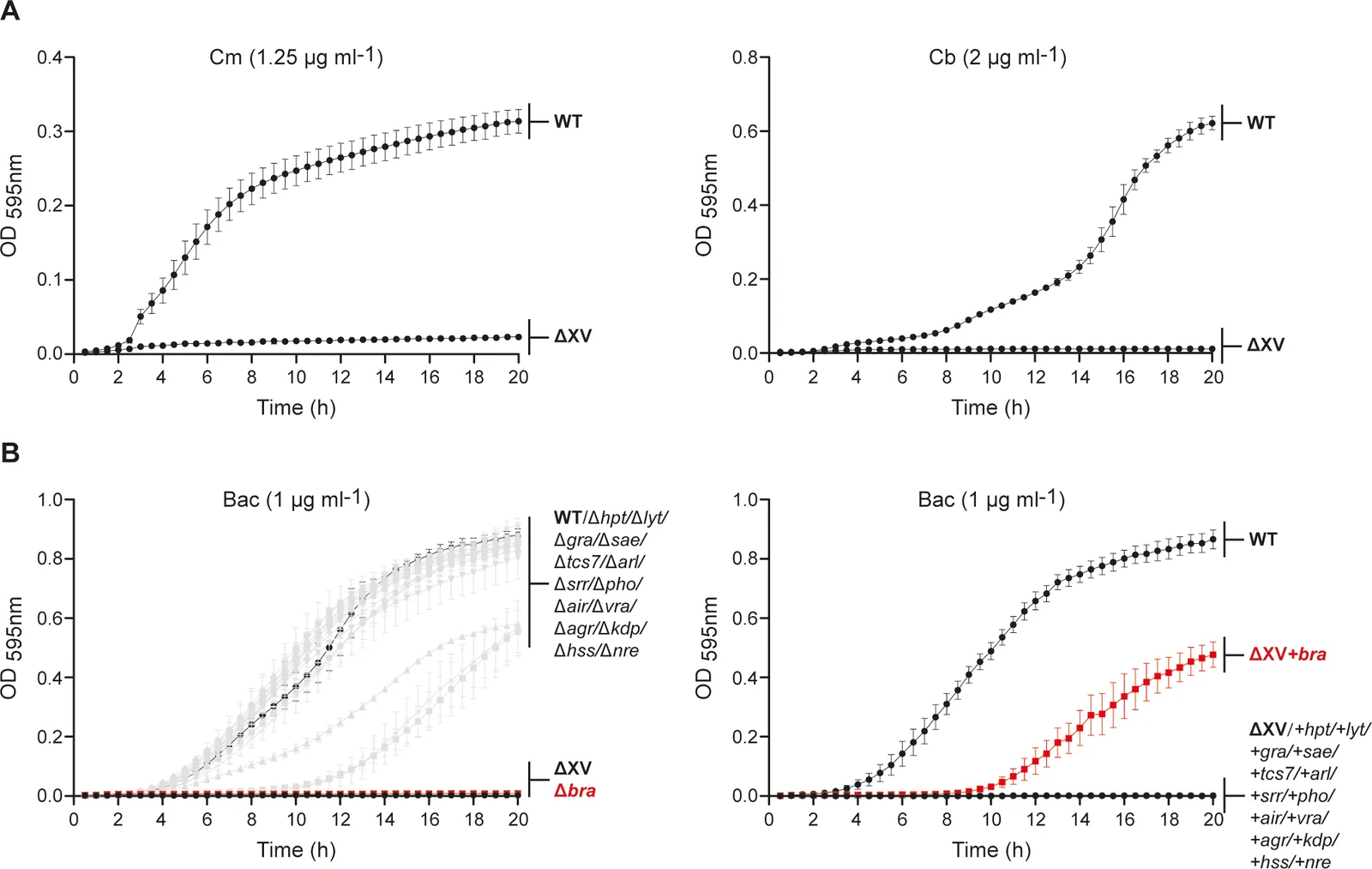
Absence of all non-essential TCSs increases *S. aureus* susceptibility to complestatin and corbomycin. (A) The optical density at 595 nm was recorded during growth of the wild-type MW2 strain and the ΔXV mutant, lacking all 15 non-essential TCSs, in MHB supplemented with Cm (left panel) or Cb (right panel) at the ΔXV MIC for 20 h at 37°C with shaking. (B) Comparison of the susceptibility to bacitracin (1 µg mL^−1^) of the wild-type MW2 strain and the ΔXV mutant with that of a collection of 15 MW2 single mutants in each non-essential TCS (left panel) and with that of a second collection of 15 strains, derivative of ΔXV, each complemented with a plasmid expressing a single TCS (right panel). In the latter case, the wild-type and ΔXV strains carried an empty pCN51 plasmid. Use of both collections allows the identification of BraRS as the specific TCS that controls *S. aureus* susceptibility to bacitracin. The average and SD of three technical replicates from one representative experiment of at least three independent experiments are shown.

To investigate if the TCS network is involved in the susceptibility of *S. aureus* to Cm and Cb, we first determined the MIC of both antibiotics for the wild-type strain MW2 and a multiple TCS mutant, MW2 ΔXV strain, lacking all 15 non-essential TCSs (36). The MIC of Cm was 2 µg mL^−1^ and 1.25 µg mL^−1^ for the wild-type and ΔXV strains, respectively, whereas the MIC of Cb was 3 µg mL^−1^ for the wild-type and 2 µg mL^−1^ for the ΔXV strain. To confirm differences in susceptibility between the wild-type and ΔXV mutant, strains were incubated in MHB supplemented with Cm or Cb at the ΔXV MIC for 20 h at 37°C, and growth was recorded over time. Results showed that, in contrast to the wild-type strain, ΔXV growth was completely halted by both antibiotics (Fig. 1A), indicating that one or various TCSs control the susceptibility to Cm and Cb in *S. aureus*.

We then used a collection of 15 single mutants in each non-essential TCS and a collection of strains, derivative of *S. aureus* ΔXV, each containing a single TCS (complete histidine kinase and response regulator pair) (Table S1). These collections are valuable tools to analyze the role of TCSs in a specific phenotype and to investigate the self-sufficiency of each TCS signaling pathway without the interference caused by other members of the network. To first validate if the use of these collections allows the identification of unique TCSs involved in *S. aureus* antibiotic resistance, we examined the susceptibility of the *S. aureus* wild type, ΔXV, and both collections of strains to the antibiotic bacitracin, which is known to be controlled mainly by the BraRS (bacitracin resistance associated) TCS. Incubation in MHB supplemented with 1 µg mL^−1^ of bacitracin for 20 h at 37°C showed that the mutant in *braRS* was the only one exhibiting sensitivity to bacitracin equal to that of ΔXV strain (Fig. 1B). Moreover, ΔXV complemented with the BraRS system was the only ΔXV derivative expressing individual TCSs showing increased resistance to bacitracin (Fig. 1B).

**Fig 2:**
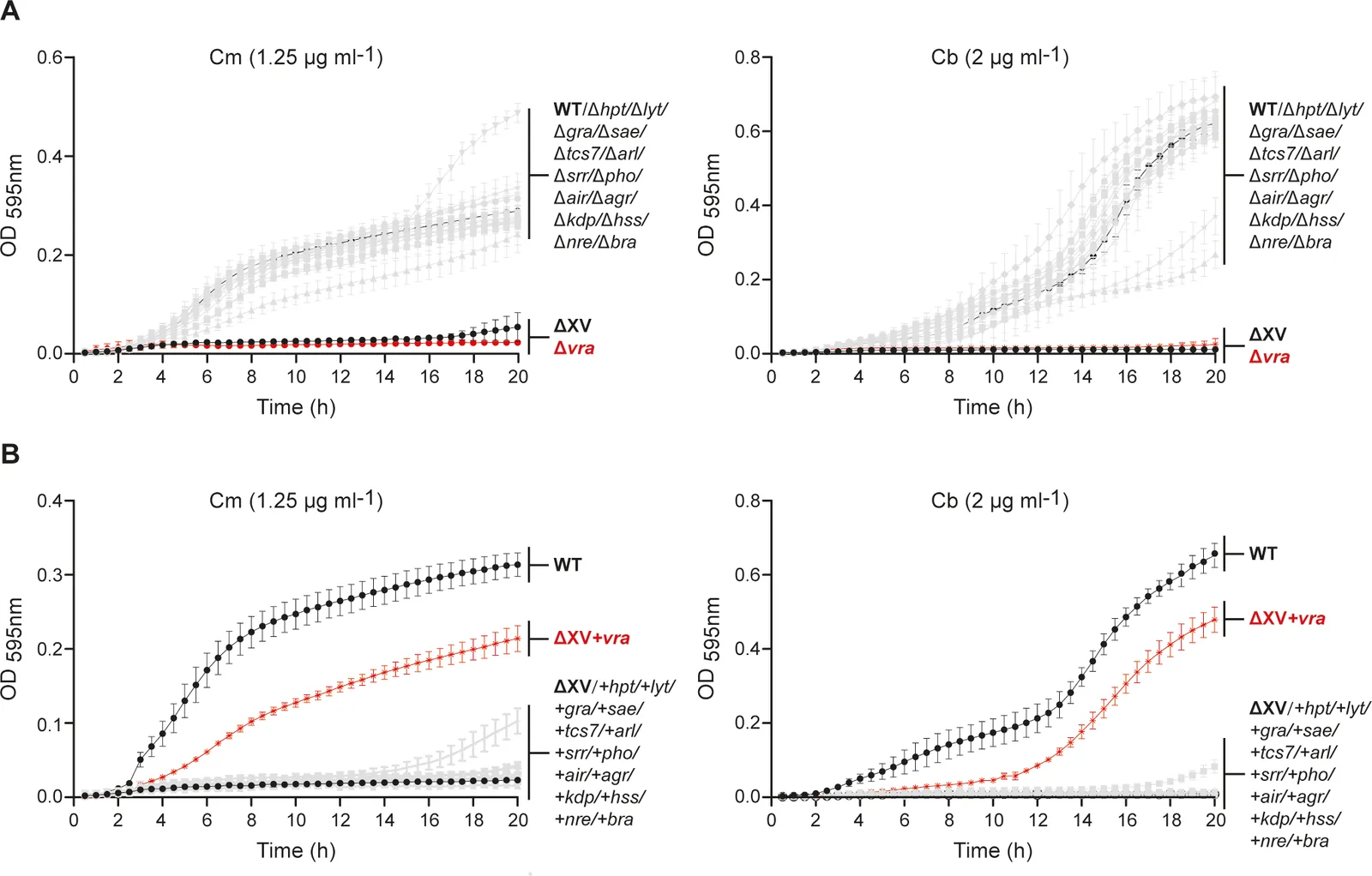
The VraSR TCS is the only non-essential TCS involved in *S. aureus* susceptibility to Cm and Cb. (A) Comparison of the susceptibility to Cm (left panel) or Cb (right panel) of the wild-type MW2 strain and the ΔXV mutant with that of a collection of 15 MW2 single mutants in each non-essential TCS. The Δ*vraSR* mutant strain is the only mutant unable to grow in the presence of the antibiotics. (B) Results obtained with a second collection of 15 strains, derivative of ΔXV, each complemented with a plasmid expressing a single TCS. In this case, the wild-type and ΔXV strains carried an empty pCN51 plasmid. The ΔXV derivative expressing *vraSR* (ΔXV + *vra*) is the only one showing a significantly increased resistance to Cm and Cb. The optical density at 595 nm was recorded during growth in MHB supplemented with the antibiotics at the ΔXV MIC for 20 h at 37°C with shaking. Average and SD of three technical replicates from one representative experiment of at least three independent experiments are shown.

Once we validated our experimental approach and to determine which TCS or TCSs is/are implicated in Cm and Cb susceptibility, we incubated the wild-type, ΔXV, and single TCS mutants in MHB under the presence of either Cm or Cb at the ΔXV MIC. The results showed that among all single mutants, Δ*vraSR* was the only one presenting a growth arrest similar to the ΔXV strain, suggesting that the VraSR TCS is the main system controlling susceptibility to both Cm and Cb (Fig. 2A). Furthermore, when the growth of the 15 ΔXV complemented strains was evaluated, increased resistance to Cm and Cb was only observed in the case of the ΔXV derivative expressing *vraSR* (Fig. 2B). Altogether, these results indicated that among all non-essential TCSs of *S. aureus*, the VraSR signal transduction pathway is the main one responsible for regulating the susceptibility to Cm and Cb, and also suggested the involvement of similar molecular mechanisms in the control of resistance to both antibiotics.

**Fig 3:**
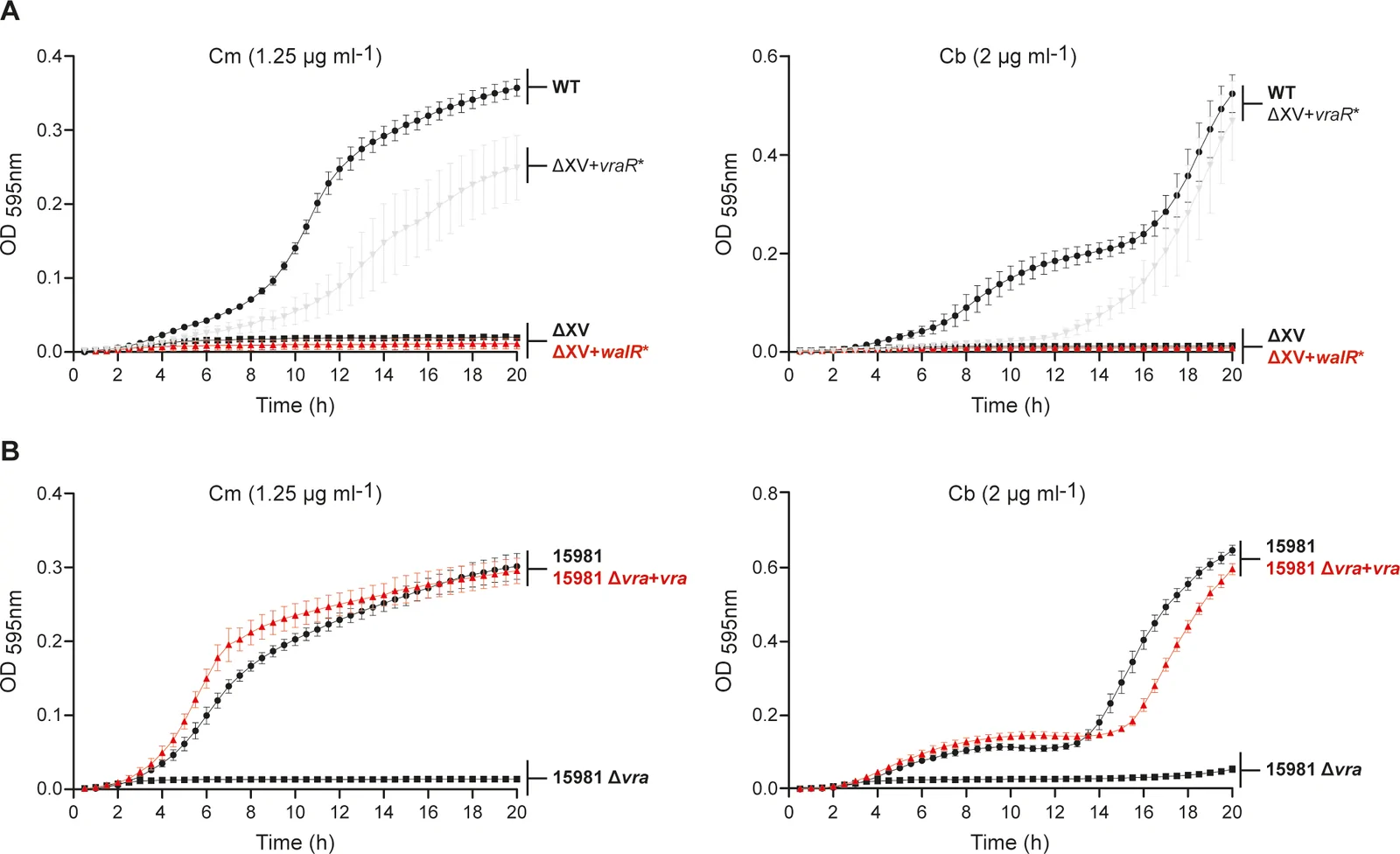
Complementation of the ΔXV mutant with a constitutively active form of WalR does not increase resistance levels of ΔXV to complestatin and corbomycin. (A) The optical density at 595 nm was recorded during growth of the wild-type MW2 strain, the ΔXV mutant, and the ΔXV strain complemented with a constitutively active form of the response regulator WalR* (WalR D52E) or the response regulator VraR* (VraR D55E), in MHB supplemented with Cm (left panel) or Cb (right panel) at the ΔXV MIC for 20 h at 37°C with shaking. The wild-type and ΔXV strains carried an empty pRMC2 plasmid. The experiments were performed in the presence of anhydrotetracycline at a concentration of 0.1 µg mL^−1^ to induce expression from the pRMC2 plasmid. The wild-type 15981 strain and the 15981 Δ*vraSR* mutant carried an empty pCN51 plasmid. The Δ*vraSR* mutant is unable to grow in the presence of both antibiotics. (B) The VraSR TCS determines Cm and Cb susceptibility in the methicillin-sensitive *S. aureus* 15981 strain. The optical density at 595 nm was recorded during growth of the wild-type 15981 strain, the 15981 Δ*vraSR* mutant, and the Δ*vraSR* mutant complemented with a pCN51 plasmid carrying the complete *vraSR* TCS (15981 Δ*vra* + *vra*), in MHB supplemented with Cm (left panel) or Cb (right panel) at the MW2 ΔXV MIC for 20 h at 37°C with shaking. Average and SD of three technical replicates from one representative experiment of at least three independent experiments are shown.

Since the ΔXV strain lacks all non-essential TCSs but maintains the *walRK* essential TCS in its genome, we analyzed a plausible role of WalRK in Cm and Cb susceptibility. To do so, we used a ΔXV strain complemented with a constitutively active form of the WalR response regulator (WalR D52E, harboring a mutation of the phosphorylation reception residue aspartic acid to a phosphomimetic residue glutamate) (Fig. 3A). Also, as a control, we analyzed a ΔXV strain complemented with a constitutively active form of VraR (VraR D55E). Overproduction of WalR D52E and VraR D55E has previously been shown to activate the WalRK and VraSR signal transduction pathways, respectively (24). As expected, the ΔXV strain expressing the constitutively active form of VraR showed enhanced resistance to Cm and Cb compared to ΔXV. On the contrary, complementation with WalR D52E did not affect Cm and Cb susceptibility.

To confirm that the role of the VraSR signaling pathway in resistance to Cm and Cb is not strain dependent and specific to the MRSA background, we tested the susceptibility of a wild-type methicillin-sensitive *S. aureus* strain (*S. aureus* 15981) (45) and its Δ*vraSR* mutant (46) to these two antibiotics (Fig. 3B). The 15981 Δ*vraSR* mutant could not grow in MHB supplemented with 1.25 µg mL^−1^ of Cm or 2 µg mL^−1^ of Cb. In contrast, complementation of the Δ*vraSR* mutant with a plasmid carrying the *vraSR* TCS restored Cm and Cb sensitivity to wild-type levels.

Overall, our results point toward VraSR as the only TCS controlling the susceptibility of *S. aureus* to the antibiotics Cm and Cb, and suggest that specific components of the VraSR regulon may be responsible for this control.

### Hypersusceptibility of the *vraSR* mutant to corbomycin and complestatin is reversed in a double *vraSR spdC* mutant

By binding to peptidoglycan, Cm and Cb block the action of autolysins, which are required to remodel the cell wall during growth (6). With this in mind and to understand how the VraSR TCS regulates the susceptibility to Cm and Cb, we analyzed the already published complete VraSR regulon looking for genes encoding proteins involved in cell-wall synthesis or remodeling and linked to autolysis (24). Overexpression of VraR D55E results in the downregulation of *ssaA*, *isaA*, and *spdC* and the upregulation of *mgt* (Table S4). Thus, higher levels of SsaA, IsaA, and SpdC and lower levels of MGT (monofunctional glycosyltransferase) are expected in a *vraSR* mutant compared to the wild-type strain. SsaA (staphylococcal secretory antigen A protein) is involved in the susceptibility to macrolide-lincosamide-streptogramin B antibiotics (47) and shows similarity to the SsaALP autolysin (48). IsaA is a putative lytic transglycosylase able to cleave peptidoglycan (49). SpdC (formerly LyrA) (50) forms a complex with the cell-wall hydrolase SagB, and the complex has been proposed to release nascent peptidoglycan strands from the cell membrane to complete integration into the cell-wall matrix (51, 52). Last, MGT is a monofunctional transglycosylase that catalyzes the elongation of peptidoglycan chains in a metal ion-dependent manner (53).

**Fig 4:**
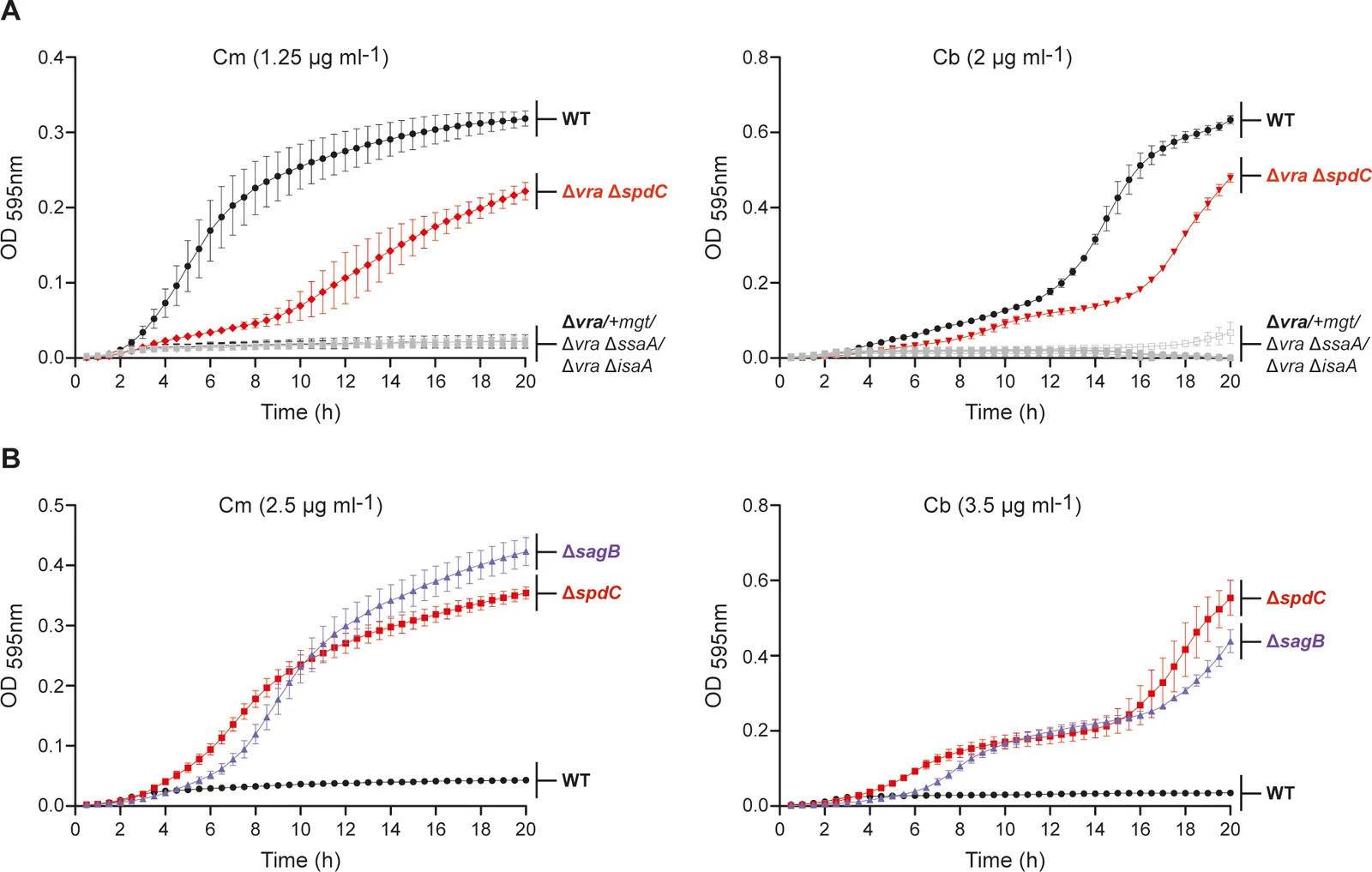
Role of SpdC and SagB in *S. aureus* susceptibility to complestatin and corbomycin. (A) Comparison of the susceptibility to Cm (left panel) or Cb (right panel) of the wild-type MW2, Δ*vraSR*, Δ*vraSR* complemented with a plasmid expressing the *mgt* gene (Δ*vra + mgt*) and the double mutants Δ*vraSR* Δ*spdC*,Δ*vraSR* Δ*ssaA*, and Δ*vraSR* Δ*isaA*. Deletion of *spdC* in the Δ*vraSR* background increases resistance to both Cm and Cb. The optical density at 595 nm was recorded during growth in MHB supplemented with Cm or Cb at the ΔXV MIC for 20 h at 37°C with shaking. (B) Comparison of the susceptibility to Cm (left panel) or Cb (right panel) of the wild-type MW2, and the single mutants Δ*spdC* and Δ*sagB*. The optical density at 595 nm was recorded during growth for 20 h, at 37°C, in MHB supplemented with Cm or Cb at a concentration above the MIC of the wild-type strain. Average and SD of three technical replicates from one representative experiment of at least three independent experiments are shown.

To determine if any of these proteins is involved in the hypersusceptible phenotype to Cm and Cb observed for the *vraSR* mutant, we constructed double mutants in *vraSR* and *ssaA*, *isaA,* or *spdC* genes, and also overexpressed *mgt* in the Δ*vraSR* background (Fig. 4A). The growth of these strains in MHB supplemented with either Cm or Cb at the ΔXV MIC showed a significant resistance recovery to both antibiotics in the case of the Δ*vraSR* Δ*spdC* mutant, while the rest of double mutants and the Δ*vraSR* overexpressing MGT were as susceptible to the antibiotics as the Δ*vraSR* mutant strain. These results indicated that abolishing SpdC production in the Δ*vraSR* mutant leads to an increase in resistance to Cm and Cb, and suggested that the hypersusceptible phenotype of Δ*vraSR* might be due to its increased SpdC levels.

### Both SpdC and SagB play a role in *S. aureus* susceptibility to Cm and Cb

SpdC is a membrane protein homolog of eukaryotic CAAX proteases that interacts with SagB, a membrane-associated *N*-acetylglucosaminidase that cleaves glycan strands of peptidoglycan to achieve the physiological length (51 – 53). By forming a complex, SpdC scaffolds SagB to orient its active site for cleaving glycan strands; thus, *spdC* and *sagB* mutants showed similar phenotypes to cell-wall-disrupting agents (resistance to lysostaphin and inhibition of teichoic acids by tunicamycin) (52). Taking this into account, we next sought to investigate the role of the SpdC/SagB complex in Cm and Cb susceptibility (Fig. 4B). To do so, we first aimed at deleting *sagB* in the Δ*vraSR* mutant to test the susceptibility to Cm and Cb of the double mutant Δ*vraSR* Δ*sagB*. However, we were unable to construct this double mutant, suggesting that VraSR and SagB are a synthetic lethal pair in *S. aureus*. Next, we constructed single mutants in *spdC* and *sagB* and evaluated their susceptibility to Cm and Cb. Notably, the MIC of Cm and Cb for both mutants raised from 2 μg mL^−1^ to 3 μg mL^−1^ and from 3 μg mL^−1^ to 4 μg mL^−1^, respectively, when compared to the wild-type strain. Accordingly, the Δ*spdC* and Δ*sagB* single mutants were able to grow in MHB supplemented with 2.5 μg mL^−1^ of Cm or 3.5 μg mL^−1^ of Cb, while the growth of the wild-type strain was inhibited under these conditions (Fig. 4B).

These results indicated that the SpdC/SagB complex is a primary determinant of Cm and Cb susceptibility *in S. aureus*.

To identify other physiological pathways that are affected by Cm and Cb, we performed a molecular evolution experiment, in which mutants with reduced susceptibility to antibiotics were selected. Among the selected mutants with moderate resistance to Cm and Cb, there were two independent mutants in the *spdC* gene and one mutant in the *vraG* gene (Table 1; Table S5). We also identified mutations in the two-component *graSR* system, three independent mutants in a protein responsible for the glycosylation of wall teichoic acid polymers (*tarS*), mutants in purine metabolism (*purR* and *purL*), and an autolysin. Importantly, many of these genes have already been linked to resistance to cationic peptides (*vraG* and *graS*) (54) and methicillin (*tarS*) (55). These results confirm the role of spdC and VraSR in susceptibility to Cm and Cb, and identify other molecular pathways related to cell-wall metabolism that also influence susceptibility to these antibiotics.

**TABLE 1:** Mutants of *S. aureus* with moderate resistance to complestatin and corbomycin^*a*^

	Complestatin		Corbomycin	
Gene	Clon 1	Clon 2	Clon 1	Clon 2
TarS	W275^*c*^	K395^*c*^		FS^*b*^ at 300/574
PurR	E167K	E167K	E167K	
PurL		Q26K		
GraS	T322K			
GraR		Q86^*c*^		
VraG			FS at 419/630	
SdpC	Δ13–23 aa		FS at 95/420	
RS08705		R119L		
FC^*b*^ MIC	4	4	2	2

^*a*^ Mutations in *S. aureus* strains selected after serial passaging at sub-MIC concentrations of antibiotic for 25 days. Passaging was performed on two independent strains. The specific sequence changes are described in Table S1.

^*b*^ FS, frame shift; FC, fold change.

^*c*^ Stop codon.

### Changes in the peptidoglycan structure provide resistance to Cm and Cb

**Fig 5:**
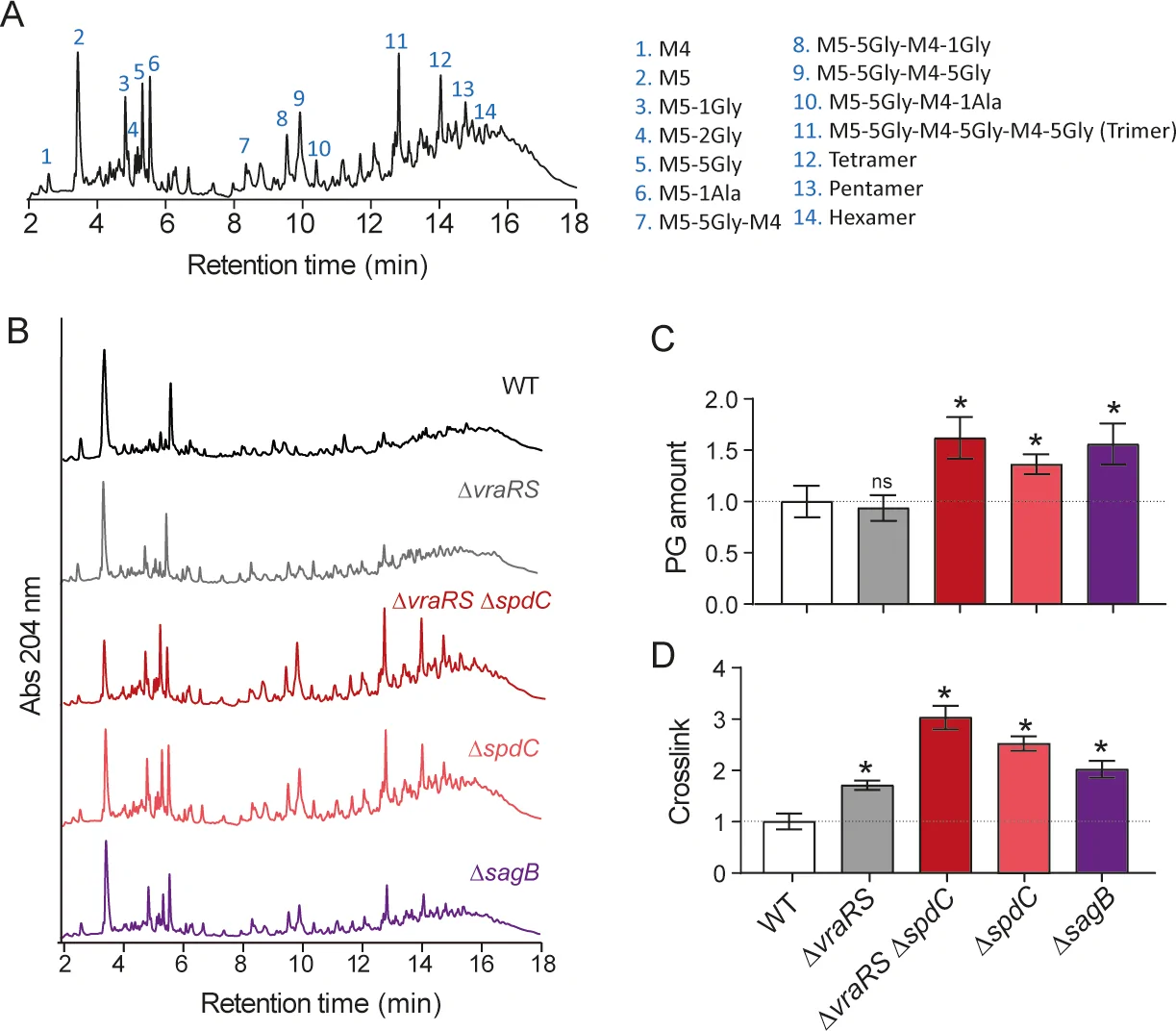
Changes in the peptidoglycan structure of *spdC* and *sagB* mutants provide resistance to Cm and Cb. (A) Peptidoglycan profile obtained for *S. aureus* MW2 *ΔspdC* mutant strain grown in TSB medium and identity of muropeptides (see Fig. S1). M, disaccharide NAG-NAM; numbers, length of stem peptides or glycine bridges. (B) Representative peptidoglycan chromatograms obtained for the wild-type, Δ*vraSR*, Δ*vraSR* Δ*spdC*, Δ*spdC*, and Δ*sagB* mutant strains grown in TSB medium. Obtained chromatograms (from three biological replicates) were used to calculate the relative amount of peptidoglycan per OD (C) and the degree of cross-linkage (D). Error bars in (C) and (D) correspond to the standard deviation of three biological replicates. ns, not significant; *P < 0.05, unpaired *t test*.

The bacteriostatic activity of Cm and Cb has been described to be the result of the binding of these molecules to the peptidoglycan and the subsequent blocking of the activity of autolysins. In this context, it is difficult to associate the lack of the hydrolase activity of the SagB autolysin with the increase of Cm and Cb resistance observed for Δ*sagB* and Δ*spdC* mutants, as a decrease in peptidoglycan hydrolase activity would be expected to exacerbate and not decrease the detrimental effect of these antibiotics. Since Cb and Cm are cell-wall-acting antibiotics, we wondered if the lack of SagB activity causes changes in the peptidoglycan structure that might confer resistance to these substances. Previous work described changes in the peptidoglycan of *sagB* mutants, but the analysis was limited to the description of the length of the glycan chains (56). To determine if there are changes in the structure of the peptidoglycan of the *sagB* and *spdC* mutants that could be responsible for the increase of resistance observed for the glycopeptide antibiotics under study, we analyzed the muropeptide composition of these mutant strains (Fig. S1). Murein sacculi were digested with muramidase, an enzyme that splits the β1–4 bonds between NAM acid and NAG producing muropeptide subunits and leaving the bridges between the peptide stems intact (57). The comparative analysis of the muropeptide profiles of the wild-type and mutant strains (Fig. 5A and B) revealed a significant increase in the relative amount of peptidoglycan per OD of culture and an increase of cross-linked muropeptides in both Δ*sagB* and Δ*spdC* strains (Fig. 5C and D). The antimicrobial activity of Cm and Cb is related to the impairment of cell-wall homeostasis. Thus, the structural changes in the peptidoglycan of *S. aureus* taking place when SagB is not active (by deleting *sagB* or its activator *spdC*) might be responsible for the increase of resistance to these glycopeptides observed in Δ*spdC* and Δ*sagB* mutants.

## DISCUSSION

In this report, we showed that VraSR is the only TCS whose absence leads to a significantly enhanced susceptibility to Cm and Cb in *S. aureus*. Two findings support this conclusion: on the one hand, when the 15 non-essential TCSs were systematically disrupted in an *S. aureus* wild-type strain, only the absence of VraSR caused a significant increase in the susceptibility to both antibiotics. On the other hand, when an *S. aureus* strain deficient in the 15 non-essential TCSs was complemented with each TCS, an almost complete restoration of the wild-type susceptibility to Cm and Cb was only achieved when *vraSR* was overexpressed. This strategy was validated using bacitracin as a query against the same two collections of strains. In agreement with previous studies, BraRS was identified as the unique TCS responsible for bacitracin susceptibility in *S. aureus* (32).

The initial report describing Cm and Cb concluded that these antibiotics block the activity of autolysins, irrespective of the enzyme family (6). *S. aureus* encodes at least 16 peptidoglycan hydrolases, including five glucosaminidases (Atl, SagA, SagB, ScaH, SsaA), two lytic transglycosylases (IsaA, SceD), four putative amidases (Atl, Sle1, LytN, EssH), and five putative endopeptidases (LytN, LytH, LytM, LytU, EssH). How these enzymes interact with each other and modulate their activity is not well understood, but it is well established that their expression needs to be tightly regulated to coordinate peptidoglycan biosynthesis and degradation to prevent cell lysis (12, 58). This regulation can occur at transcriptional and post-translational levels through protein-protein interactions, conformational changes, and structural dynamics that affect the activity of PG synthesis enzymes (59). At the transcriptional level, several *S. aureus* TCSs strongly contribute to regulating autolysins expression. For instance, WalRK positively controls the expression of Atl, LytM, SsaA, IsaA, SceD, EssH, and various CHAP (Cysteine, Histidine-dependent Amidohydrolases/Peptidases)-containing putative peptidoglycan hydrolases (SA0620, SA2097, and SA2353) (23, 60). Consequently, cells without WalRK have reduced peptidoglycan hydrolytic activity but continued peptidoglycan synthesis (60). GraSR also positively regulates the expression of Atl, AsaA, IsaA, ScdE, and various CHAP-containing putative peptidoglycan hydrolases (SA2353, SA0620, SA2097, and SA2332) (61). ArlRS represses the expression of LytN and EssH (62, 63). Other TCSs, such as AgrCA and SrrAB, also control the autolysis rate by regulating the expression of LytM and IsaA, respectively (49). A special case is LytRS, which negatively regulates peptidoglycan hydrolytic activity by regulating the expression of the *lrgAB* operon, which apparently controls the transport of autolysins across the membrane (64). Despite all these TCSs having a profound effect on the regulation of the expression and/or activity of several autolysins, in the present study, we have demonstrated that their deletion or constitutive activation, in the case of WalR, does not significantly affect the susceptibility of *S. aureus* to Cm and Cb and that VraSR is the only TCS responsible for tolerance to both antibiotics. The VraSR regulon was originally identified by comparing the transcriptional response of a wild-type and its isogenic *vraSR* mutant strain after vancomycin treatment (65). The results revealed that VraSR regulates several genes associated with cell-wall peptidoglycan synthesis, such as *pbp2*, *sgtB*, *murZ*, *fmtA*, and teicoplanin-resistance-related proteins (*tcaA*/*tcaB*). More recently, we elucidated all TCSs’ regulons by complementing a strain devoid of the complete non-essential TCS network (ΔXV mutant) with the constitutively active form of each response regulator. Results established that three autolysins were downregulated (*ssaA*, *isaA*, and *spdC*/*sagB*), whereas one of them (*mgt*) was upregulated in the ΔXV strain overexpressing VraR D55E (24). Interestingly, all these autolysins were also shown to be regulated by at least another TCS, while deletion of *spdC* was enough to restore the susceptibility of the *vraSR* mutant to Cm and Cb to almost wild-type levels. SpdC is an integral membrane protein that forms a complex with SagB and orients the active site of SagB for efficient cleavage of the nascent peptidoglycan to produce free oligomers that can undergo further peptidoglycan elongation. As *spdC* and *sagB* mutants are functionally related, we made several attempts to mutate *sagB* in the *vraSR* mutant. However, we were unable to generate the double *vraSR*/*sagB* mutant. Thus, we generated single mutants in *spdC* and *sagB*. Both single mutants showed decreased susceptibility to Cm and Cb compared to the wild-type strain. All in all, these findings do not allow us to firmly conclude that the greater susceptibility to Cm and Cb shown by the *vraSR* mutant is due to the overproduction of SpdC, but they reveal that *spdC* and/or *sagB* deficiency decreases *S. aureus* susceptibility to both antibiotics. Further evidence for the contribution of SpdC/SagB to Cd and Cm susceptibility comes from *in vitro* evolutionary experiments in which independent mutants in SpdC were selected. The same experiment identifies GraSR and VraG mutants with increased antibiotic resistance. The selection of these mutants is interesting because it has been described that the interaction of VraG with GraS is necessary for GraS to recognize cationic peptides (54).

While it might seem contradictory that the lack of activity of SagB autolysin recovers the resistance to the autolysin-inhibiting antibiotics Cm and Cb, certain autolysins have been previously reported to strengthen the bactericidal or bacteriostatic activity of other antibiotics (8). The structural changes observed in the cell wall when SagB is inactive (i.e., increase in the relative amount of peptidoglycan per OD of culture and cross-linkage) could help to maintain cell-wall homeostasis in the presence of Cm and Cb, providing resistance to these antibiotics. For the specific case of Cm and Cb, remodeling the peptidoglycan structure might directly interfere with the activity of other autolysins: *S. aureus* hypocross-linked peptidoglycans lead to an increased *in vitro* hydrolysis of cell walls by autolytic enzymes (66), and some peptidoglycan hydrolases have been described to present substrate specificity (39). Additionally, although in our experiments we did not see a significant decrease in the relative amount of peptidoglycan in the Δ*vraSR* mutant in comparison with the wild-type strain (Fig. 5C), VraSR has been shown to positively regulate cell-wall peptidoglycan synthesis (65). In this scenario, the hypersusceptibility to Cm and Cb of the ΔvraRS mutant could also be alleviated by the increase in peptidoglycan resulting from the deletion of SpdC or SagB. It remains uncertain whether these effects are direct or instead affect specific downstream cell-wall components. On the other hand, decreased transcription and enzymatic activities of several key autolytic enzymes of *S. aureus* lead to a reduction of autolysis induced by some cell-wall-acting antibiotics, thus the single loss of function of the SagB autolysin may also be behind the Cm and Cb resistance phenotype observed for *spdC* and *sagB* mutants. Future investigations should aim to elucidate the mechanistic role of SagB and SpdC in antibiotic resistance.

### Conclusions

These data contribute to our understanding of which TCS is responsible for controlling homeostatic cell-wall remodeling that is perturbed by the presence of Cm and Cb. Several TCSs monitor the correct expression of autolysins to avoid either insufficient activity that would prevent cell-wall expansion or excessive activity that would cause lysis of the bacteria. Our results indicate that the regulatory activity of TCSs does not overlap. The VraSR system is the only TCS whose absence affects the susceptibility of *S. aureus* to these antibiotics. This phenotype is related to the overproduction of SpdC and its regulatory activity on SagB. However, other VraRS regulon genes must be involved in this phenotype because SpdC expression is controlled by other TCSs, and still only the absence of VraRS affects Cm and Cb susceptibility.

We can envision at least two limitations in this study. Some TCSs may not be in the phosphorylated state under the tested conditions. It is important to note that the cognate signals for most *S. aureus* TCSs are still unknown. This opens the possibility that other TCSs might also affect the susceptibility to Cm and Cb. We did not consider phosphorylation through serine/threonine kinases and phosphatases, which play major roles in regulating cell-wall synthesis and antibiotic susceptibility. *S. aureus* has one serine/threonine kinase (Stk1 or PknB) and a cognate phosphatase Stp1. These questions should be addressed in future studies.

## SUPPLEMENTAL MATERIAL

Supplemental material spectrum.00370-23-s0001.docx This file includes Fig. S1 and Tables S1 to S5.

ASM does not own the copyrights to Supplemental Material that may be linked to, or accessed through, an article. The authors have granted ASM a non-exclusive, world-wide license to publish the Supplemental Material files. Please contact the corresponding author directly for reuse.
