# Supplementary material for: Staphylococcus aureus susceptibility to complestatin and corbomycin depends on the VraSR two-component system
Source: Microbiol Spectr. 2023 Aug 30;11(5):e00370-23. doi: 10.1128/spectrum.00370-23 (PMC10581084; doi:10.1128/spectrum.00370-23)
Supplement: Supplemental material — This file includes Fig. S1 and Tables S1 to S5. [file spectrum.00370-23-s0001.docx]

***Supplementary material***

***Staphylococcus aureus* susceptibility to corbomycin and complestatin depends on the VraSR two component system**

Carmen Gómez-Arrebola^1^, Sara B. Hernandez^2^, Elizabeth J. Culp^3^, Gerard D. Wright^3^, Cristina Solano^1^, Felipe Cava^2#^ and Iñigo Lasa^1#^

^1^ Laboratory of Microbial Pathogenesis. Navarrabiomed, Hospital Universitario de Navarra (HUN), Universidad Pública de Navarra (UPNA), IdiSNA, Pamplona-31008, Spain.

^2^ Laboratory for Molecular Infection Medicine Sweden, Department of Molecular Biology, Umeå Centre for Microbial Research, Umeå University, Umeå SE-90187, Sweden.

^3^ M. G. DeGroote Institute for Infectious Disease Research, David Braley Centre for Antibiotic Discovery, Department of Biochemistry and Biomedical Sciences, McMaster University, Hamilton, Ontario, Canada

^#^ Address correspondence to Iñigo Lasa and Felipe Cava

E-mail address: [ilasa@unavarra.es](mailto:ilasa@unavarra.es) ; felipe.cava@umu.se

**This file includes:**

Supplementary Tables and figures

References

**Table S1.** Strains used in this study

| Strains | Relevant characteristics | Lab number | Reference |
| --- | --- | --- | --- |
| *Staphylococcus aureus* |  |  |  |
| MW2 | Community-acquired methicillin-resistant *Staphylococcus aureus* (CA-MRSA) | 3566 | (1) |
| ΔXV | MW2 ΔXV | 2961 | (2) |
| Δ*hpt* | MW2 Δ*hptSR* | 4032 | (2) |
| Δ*lyt* | MW2 Δ*lytSR* | 2964 | (2) |
| Δ*gra* | MW2 Δ*graRS* | 11 | (2) |
| Δ*sae* | MW2 Δ*saeRS* | 2965 | (2) |
| Δ*tcs7* | MW2 Δ*MW1208-MW1209* | 4033 | (2) |
| Δ*arl* | MW2 Δ*arlRS* | 4034 | (2) |
| Δ*srr* | MW2 Δ*srrAB* | 2966 | (2) |
| Δ*pho* | MW2 Δ*phoPR* | 4035 | (2) |
| Δ*air* | MW2 Δ*airSR* | 3670 | (2) |
| Δ*vra* | MW2 Δ*vraSR* | 4036 | (2) |
| Δ*agr* | MW2 Δ*agrBDCA* | 4037 | (2) |
| Δ*kdp* | MW2 Δ*kdpDE* | 4038 | (2) |
| Δ*hss* | MW2 Δ*hssRS* | 2979 | (2) |
| Δ*nre* | MW2 Δ*nreBC* | 2967 | (2) |
| Δ*bra* | MW2 Δ*braRS* | 4039 | (2) |
| MW2 pCN51 | MW2 carrying pCN51 empty plasmid | 4681 | (2) |
| ΔXV pCN51 | MW2 ΔXV carrying pCN51 empty plasmid | 4682 | (2) |
| ΔXV + *hpt* | MW2 ΔXV carrying pCN51::*hptSR* plasmid | 5232 | This study |
| ΔXV + *lyt* | MW2 ΔXV carrying pCN51::*lytSR* plasmid | 5239 | This study |
| ΔXV + *gra* | MW2 ΔXV carrying pCN51::*graRS* plasmid | 5270 | (2) |
| ΔXV + *sae* | MW2 ΔXV carrying pCN51::*saeRS* plasmid | 5233 | This study |
| ΔXV + *tcs7* | MW2 ΔXV carrying pCN51::*MW1208-1209* plasmid | 5241 | This study |
| ΔXV + *arl* | MW2 ΔXV carrying pCN51::*arlRS* plasmid | 4538 | (2) |
| ΔXV + *srr* | MW2 ΔXV carrying pCN51::*srrAB* plasmid | 4680 | (2) |
| ΔXV + *pho* | MW2 ΔXV carrying pCN51::*phoPR* plasmid | 5234 | This study |
| ΔXV + *air* | MW2 ΔXV carrying pCN51::*airSR* plasmid | 5235 | This study |
| ΔXV + *vraS* | MW2 ΔXV carrying pCN51::*vraSR* plasmid | 4676 | (2) |
| ΔXV + *agr* | MW2 ΔXV carrying pCN51::*agrBDCA* plasmid | 5236 | This study |
| ΔXV + *kdp* | MW2 ΔXV carrying pCN51::*kdpDE* plasmid | 5264 | This study |
| ΔXV + *hss* | MW2 ΔXV carrying pCN51::*hssRS* plasmid | 5237 | This study |
| ΔXV + *nre* | MW2 ΔXV carrying pCN51::*nreBC* plasmid | 4536 | (2) |
| ΔXV + *bra* | MW2 ΔXV carrying pCN51::*braRS* plasmid | 5238 | This study |
| Δ*vra* + *vra* | MW2 Δ*vraSR* carrying pCN51::*vraSR* plasmid | 4675 | (2) |
| 15981 pCN51 | 15981 carrying pCN51 empty plasmid | 4814 | This study |
| 15981 Δ*vra* pCN51 | 15981 Δ*vraSR*  carrying pCN51 empty plasmid | 8143 | This study |
| 15981 Δ*vra* + *vra* | 15981 Δ*vraSR* carrying pCN51::*vraSR* plasmid | 8144 | This study |
| MW2 pRMC2 | MW2 carrying pRMC2 empty plasmid | 6870 | (3) |
| ΔXV pRMC2 | MW2 ΔXV carrying pRMC2 empty plasmid | 6871 | (3) |
| ΔXV + *vraR** | MW2 ΔXV carrying pRMC2::*vraR*D55E plasmid | 6882 | (3) |
| ΔXV + *walR** | MW2 ΔXV carrying pRMC2::*walR*D52E plasmid | 6872 | (3) |
| Δ*vra* Δ*ssaA* | MW2 Δ*vraRS* Δ*ssaA* | 8229 | This study |
| Δ*vra* Δ*isaA* | MW2 Δ*vraRS* Δ*isaA* | 8230 | This study |
| Δ*vra* Δ*spdC* | MW2 Δ*vraRS* Δ*spdC* | 8228 | This study |
| Δ*vra* + *mgt* | MW2 Δ*vraSR* carrying pCN51::*mgt* plasmid | 8189 | This study |
| Δ*spdC* | MW2 Δ*spdC* | 8344 | This study |
| Δ*sagB* | MW2 Δ*sagB* | 8433 | This study |
| *Escherichia coli* |  |  |  |
| *E. coli* IM01B | *E. coli* K12 DH10B Δ*dcm* and containing the *hsdS* gene of MW2 integrated between the *essQ* and *cspB* genes. Used for cloning experiments and isolation of plasmids that are transformed into *S. aureus* CC1 strains at high efficiency. | 5694 | (4) |

^a^ Number of each strain in the culture collection of the Laboratory of Microbial Pathogenesis, Navarrabiomed-Universidad Pública de Navarra.

**Table S2.** Plasmids used in this study

| **Plasmids** | **Relevant characteristics** | **Reference** |
| --- | --- | --- |
| pJET1.2 | Cloning vector. Amp^R^ | Thermo Scientific |
| pCN51 | *E. coli* - *S. aureus* shuttle vector to express genes under the control of the P_cad_ cadmium-inducible promoter. Low copy number (20 to 25 copies/cell). Ery^R^ | (5) |
| pCN51::*hptRS* | pCN51 plasmid expressing *hptRS* genes | This study |
| pCN51::*lytRS* | pCN51 plasmid expressing *lytRS* genes | This study |
| pCN51::*graRS* | pCN51 plasmid expressing *graRS* genes | (2) |
| pCN51::*saeRS* | pCN51 plasmid expressing *saeRS* genes | This study |
| pCN51::*tcs7RS* | pCN51 plasmid expressing *tcs7RS* genes | This study |
| pCN51::*arlRS* | pCN51 plasmid expressing *arlRS* genes | (2) |
| pCN51::*srrAB* | pCN51 plasmid expressing *srrAB* genes | (2) |
| pCN51::*phoRP* | pCN51 plasmid expressing *phoRP* genes | This study |
| pCN51::*airRS* | pCN51 plasmid expressing *airRS* genes | This study |
| pCN51::*vraRS* | pCN51 plasmid expressing *vraRS* genes | (2) |
| pCN51::*agrCA* | pCN51 plasmid expressing *agrRS* genes | This study |
| pCN51::*kdpDE* | pCN51 plasmid expressing *kdpDE* genes | This study |
| pCN51::*hssRS* | pCN51 plasmid expressing *hssRS* genes | This study |
| pCN51::*nreBC* | pCN51 plasmid expressing *nreBC* genes | (2) |
| pCN51::*braRS* | pCN51 plasmid expressing *braRS* genes | This study |
| pCN51::*mgt* | pCN51 plasmid expressing the *mgt* gene | This study |
| pRMC2 | Anhydrotetracycline-inducible expression plasmid. Clo^R^ | (6) |
| pRMC2::*walR*D52 | pRMC2 plasmid expressing the phosphomimetic form of WalR | (3) |
| pRMC2::*vraR*D55E | pRMC2 plasmid expressing the phosphomimetic form of VraR | (3) |
| pMAD | *E. coli* - *S. aureus* shuttle vector containing a thermosensitive origin of replication for Gram-positive bacteria. Amp^R^ Ery^R^ | (7) |
| pMAD::*ssaA* | pMAD plasmid containing the sequence for deletion of the *ssaA* coding sequence | This study |
| pMAD::*isaA* | pMAD plasmid containing the sequence for deletion of the *isaA* coding sequence | This study |
| pMAD::*spdC* | pMAD plasmid containing the sequence for deletion of the *spdC* coding sequence | This study |
| pMAD::*sagB* | pMAD plasmid containing the sequence for deletion of the *sagB* coding sequence | This study |

**Table S3.** Oligonucleotides used in this study

| Oligonucleotide | Sequence^a^ |
| --- | --- |
| Constitutive expression of TCSs | |
| TCS3S_Rv (XmaI AscI) | GGCGCGCCCGGGtaccttaaacatctacattc |
| TCS3S_Fw (BamHI) | GGATCCtttttggagatgattcaatg |
| TCS3R_Rv (BamHI XmaI KpnI) | GGTACCAATACCCGGGCAATGGATCCaatctattttgcttgcttac |
| TCS3R_Fw (XhoI SalI) | GTCGACATTGCTCGAGttcaagggggaatgtagat |
| LytS_Rv (XmaI AscI) | GGCGCGCCCGGGtatttattcctcctcttgtc |
| LytS_Fw (BamHI) | GGATCCaatttactgaggtgctatcg |
| LytR_Rv (BamHI XmaI KpnI) | GGTACCAATACCCGGGCAATGGATCCactgttaaagtaaccctatc |
| LytR_Fw (XhoI SalI) | GTCGACATTGCTCGAGgacaagaggaggaataaata |
| SaeS_Rv (XmaI AscI) | GGCGCGCCCGGGatcggattatgacgtaatgt |
| SaeS_Fw (BamHI) | GGATCCatttgaaaggagccgataat |
| SaeR_Rv (BamHI XmaI KpnI) | GGTACCAATACCCGGGCAATGGATCCattatcggctcctttcaaat |
| SaeR_Fw (XhoI SalI) | GTCGACATTGCTCGAGcgaacagaggtgaaaaaatag |
| TCS7S_Rv (XmaI AscI) | GGCGCGCCCGGGaaagatgtcatgctattcct |
| TCS7S_Fw (BamHI) | GGATCCaaagggcggaataaaatatg |
| TCS7R_Rv (BamHI XmaI KpnI) | GGTACCAATACCCGGGCAATGGATCCatttagatccagcctttttc |
| TCS7R_Fw (XhoI SalI) | GTCGACATTGCTCGAGaacaggaggaatagcatga |
| PhoR(S)_Rv (XmaI AscI) | GGCGCGCCCGGGttttattctttataatcttttag |
| PhoR(S)_Fw (BamHI) | GGATCCattggaaagacctaaagaac |
| PhoP(R)_Rv (BamHI XmaI KpnI) | GGTACCAATACCCGGGCAATGGATCCtcatcattgttctttaggtc |
| PhoP(R)_Fw (XhoI SalI) | GTCGACATTGCTCGAGataagttagggaggcatac |
| AirS_Rv (XmaI AscI) | GGCGCGCCCGGGGctattttataggaattgtg |
| AirS_Fw (BamHI) | GGATCCaaatgaattggagcgatttg |
| AirR_Rv (BamHI XmaI KpnI) | GGTACCAATACCCGGGCAATGGATCCttctaaatcaacttattttcc |
| AirR_Fw (XhoI SalI) | GTCGACATTGCTCGAGatttaaggagataacccatg |
| AgrC(S)_Rv (XmaI AscI) | GGCGCGCCCGGGGGctagttgttaataatttc |
| AgrC(S)_Fw (BamHI) | GGATCCtataagagaaagtgtgatag |
| AgrA(R)_Rv (BamHI XmaI KpnI) | GGTACCAATACCCGGGCAATGGATCCtatcttattatatttttttaacg |
| AgrA(R)_Fw (XhoI SalI) | GTCGACATTGCTCGAGcataaggatgtgaatgtatg |
| KdpD(S)_Rv (XmaI AscI) | GGCGCGCCCGGGcattatacgtctccttcatt |
| KdpD(S)_Fw (BamHI) | GGATCCtatcgaggtgaaggttatg |
| KdpE(R)_Rv (BamHI XmaI KpnI) | GGTACCAATACCCGGGCAATGGATCCattatttctctttccactgc |
| KdpE(R)_Fw (XhoI SalI) | GTCGACATTGCTCGAGaatgaaggagacgtataatg |
| HssS_Rv (XmaI AscI) | GGCGCGCCCGGGagattaaagtgaattatttgg |
| HssS_Fw (BamHI) | GGATCCcaaggctataaggtggaga |
| HssR_Rv (BamHI XmaI KpnI) | GGTACCAATACCCGGGCAATGGATCCtttaaacatgattctccacc |
| HssR_Fw (XhoI SalI) | GTCGACATTGCTCGAGgataagggagtttatagcta |
| BraS_Rv (XmaI AscI) | GGCGCGCCCGGGtttttattcatctggaaattg |
| BraS_Fw (BamHI) | GGATCCagtatagggtgaatgcaatg |
| BraR_Rv (BamHIXmaI KpnI) | GGTACCAATACCCGGGCAATGGATCCcctatactttatatccgaca |
| BraR_Fw (XhoI SalI) | GTCGACATTGCTCGAGttgaaggaagaagattatag |
| Overexpression of *mgt* |  |
| mgt_Fw | ggGGATCCtaaactcaaggtatatactaagtgag |
| mgt_Rv | ggGAATTCttaacgatttaattgtgacatagc |
| Deletion of genes that are part of the VraRS regulon | |
| ssaA_A | ggAGATCTaaactagttgatcgtgctg |
| ssaA_B | tatcatcgtgtttatagaataaagtcctccaaagttc |
| ssaA_C | gaggactttattctataaacacgatgatacatattgtc |
| ssaA_D | ggGAATTCacctttaattaaagtgtctttatcg |
| ssaA_E | gcaaatccaatgatgagaac |
| isaA_A | ggAGATCTgatatttttaaattcatgggataataac |
| isaA_B | tatttattatgaaggaaagtaaaaaatcctccagtaataattg |
| isaA_C | gattttttactttccttcataataaataaaagtaatgtttag |
| isaA_D | ggGAATTCcaacaaacatttttttaagtgca |
| isaA_E | gcttgcagatatcatttatcg |
| spdC_A | ggCCATGGttgtttaaagcaacacctttg |
| spdC_B | gctttgttatatatgtaacctccattaggtaac |
| spdC_C | ggttacatatataacaaagcgcttgctagtac |
| spdC_D | ggGGATCCgtgctgatgaagtagatcc |
| spdC_E | gcaaatgcttgtttaatcg |
| sagB_A | ggAGATCTggtaacaatggattacgcac |
| sagB_B | aactctatcaaaatccacacctcttaggtc |
| sagB_C | ctaagaggtgtggattttgatagagttaagttatgatataacttg |
| sagB_D | ggGAATTCgtatatcgtcgtattcggc |
| sagB_E | gcgcaccttggaatatag |

^a^ Restriction enzymes sites are indicated in upper case.

**Table S4.** Expression levels of VraSR regulated genes encoding proteins involved in cell wall assembly and linked to autolysis

| Locus Tag | | Gene | RNA Seq log_2_ Fold Change^a^ | FDR corrected p-value | Fold Change |
| --- | --- | --- | --- | --- | --- |
| MW_RS12065 | MW2222 | *ssaA* | -2.037695 | 0.00072695 | -4.1 |
| MW_RS13490 | MW2490 | *isaA* | -1.2586757 | 0.0105 | -2.4 |
| MW_RS12230 | MW2255 | *spdC* | -1.0553137 | 0.0140455 | -2.1 |
| MW_RS09880 | MW1814 | *mgt* | 1.50374561 | 1.40E^-06^ | 2.8 |

^a^RNA seq data extracted from (3)Differential gene expression data was obtained after comparing the MW2 pRMC2::vraR D55E samples (constitutively active form of VraR) with the MW2 pRMC2 control samples.

**Table S5.** Specific sequence changes in selected *S. aureus* mutants with moderate resistance to complestatin and corbomycin

| Comp | | Corb | |  |  |  |  |  |
| --- | --- | --- | --- | --- | --- | --- | --- | --- |
| A | B | A | B | Position | DNA mutation | Translational change | Gene name | Annotation |
|  |  |  |  | 128537 | G→A | W275* (TGG→TGA) | *tarS* | glycosyltransferase family 2 protein |
|  |  |  |  | 87565 | C→T | A43V (GCT→GTT) | *SAUPAN006240000* | TetR/AcrR family transcriptional regulator |
|  |  |  |  | 128612 | +A | coding (900/1722 nt) | *tarS* | glycosyltransferase family 2 protein |
|  |  |  |  | 128895 | A→T | K395* (AAG→TAG) | *tarS* | glycosyltransferase family 2 protein |
|  |  |  |  | 14257 | G→A | E167K (GAA→AAA) | *purR* | pur operon repressor |
|  |  |  |  | 91300 | C→T | Q86* (CAA→TAA) | *graR* | DNA‑binding response regulator |
|  |  |  |  | 92676 | C→A | T322K (ACA→AAA) | *graS* | sensor histidine kinase |
|  |  |  |  | 94903 | (A)6→5 | coding (1257/1890 nt) | *vraG* | bacitracin ABC transporter permease |
|  |  |  |  | 184069 | C→A | Q26K (CAA→AAA) | *purL* | phosphoribosylformylglycinamidine synthase subunit PurL |
|  |  |  |  | 157210 | C→A | R119L (CGT→CTT) | *SAUPAN004467000* | autolysin |
|  |  |  |  | 2313 | Δ30 bp | coding (40‑69/1260 nt) | *spdC* | lysostaphin resistance protein A |
|  |  |  |  | 2558 | +T | coding (285/1260 nt) | *spdC* | lysostaphin resistance protein A |
|  |  |  |  | 44011 | N→C | ?807D (GAN→GAC) | *sdrC* | hydrolase |

**Figure S1.** Mass analysis of *S. aureus* muropeptides. (A) Table presenting the data obtained in the mass analysis performed for muropeptide identification on the muramidase digested peptidoglycan of *S. aureus* MW2 *ΔspdC* mutant strain grown in TSB medium (Figure 5A)*.* (B) Schematic representation of identified muropeptides. NAG: *N*-acetyl-glucosamine; NAM: *N*-acetyl-muramic acid; M: disaccharide NAG-NAM; numbers: length of peptides; D and L indicate the amino acids isomer; Ala: alanine; Gln: glutamine; Lys: lysine; Gly: glycine.

**
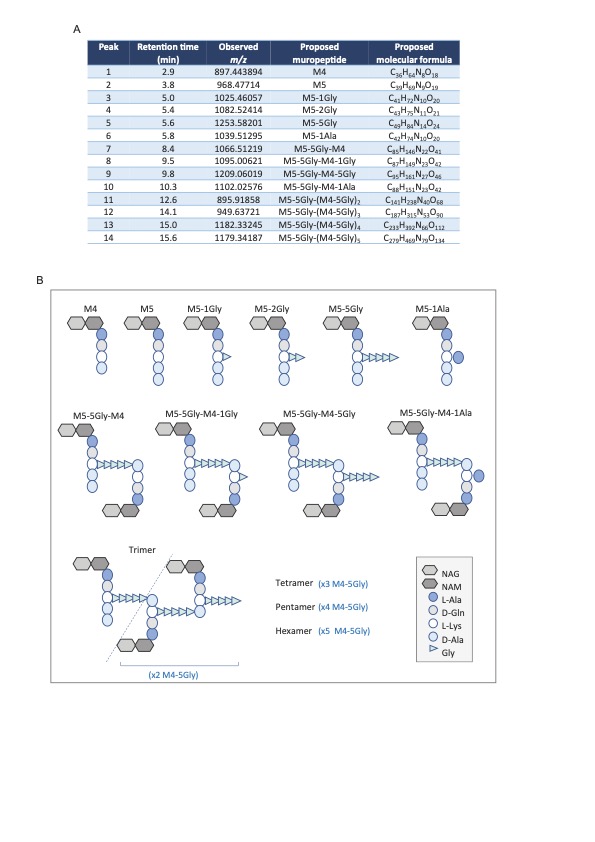
**

**Supplementary References**

1. Baba T, Takeuchi F, Kuroda M, Yuzawa H, Aoki K, Oguchi A, Nagai Y, Iwama N, Asano K, Naimi T, Kuroda H, Cui L, Yamamoto K, Hiramatsu K. 2002. Genome and virulence determinants of high virulence community-acquired MRSA. Lancet 359:1819–1827.

2. Villanueva M, García B, Valle J, Rapún B, Mozos IR de los, Solano C, MartI M, Penadés JR, Toledo-Arana A, Lasa I. 2018. Sensory deprivation in *Staphylococcus aureus*. Nat Commun 9:523.

3. Rapun-Araiz B, Haag AF, Cesare VD, Gil C, Dorado-Morales P, Penades JR, Lasa I. 2020. Systematic Reconstruction of the Complete Two-Component Sensorial Network in *Staphylococcus aureus*. mSystems 5:e00511-20.

4. Monk IR, Tree JJ, Howden BP, Stinear TP, Foster TJ. 2015. Complete bypass of restriction systems for major *Staphylococcus aureus* lineages. mBio 6:1–12.

5. Charpentier E, Anton AI, Barry P, Alfonso B, Fang Y, Novick RP. 2004. Novel cassette-based shuttle vector system for gram-positive bacteria. Appl Environ Microbiol 70:6076–6085.

6. Corrigan RM, Foster TJ. 2009. An improved tetracycline-inducible expression vector for *Staphylococcus aureus*. Plasmid 61:126–129.

7. Arnaud M, Chastanet A, Débarbouillé M. 2004. New vector for efficient allelic replacement in naturally nontransformable, low-GC-content, gram-positive bacteria. Appl Environ Microbiol 70:6887–6891.
